# Potential of Start Codon Targeted (SCoT) Markers to Estimate Genetic Diversity and Relationships among Chinese *Elymus sibiricus* Accessions

**DOI:** 10.3390/molecules20045987

**Published:** 2015-04-07

**Authors:** Junchao Zhang, Wengang Xie, Yanrong Wang, Xuhong Zhao

**Affiliations:** The State Key Laboratory of Grassland Agro-ecosystems, College of Pastoral Agriculture Science and Technology, Lanzhou University, Lanzhou 730020, China; E-Mails: 13669321907@163.com (J.Z.); zhaoxh14@lzu.edu.cn (X.Z.)

**Keywords:** *Elymus sibiricus*, SCoT marker, genetic diversity, conservation

## Abstract

*Elymus sibiricus* as an important forage grass and gene pool for improving cereal crops, that is widely distributed in West and North China. Information on its genetic diversity and relationships is limited but necessary for germplasm collection, conservation and future breeding. Start Codon Targeted (SCoT) markers were used for studying the genetic diversity and relationships among 53 *E. sibiricus* accessions from its primary distribution area in China. A total of 173 bands were generated from 16 SCoT primers, 159 bands of which were polymorphic with the percentage of polymorphic bands (PPB) of 91.91%. Based upon population structure analysis five groups were formed. The cluster analysis separated the accessions into two major clusters and three sub-clusters, similar to results of principal coordinate analysis (PCoA). The molecular variance analysis (AMOVA) showed that genetic variation was greater within geographical regions (50.99%) than between them (49.01%). Furthermore, the study also suggested that collecting and evaluating *E. sibiricus* germplasm for major geographic regions and special environments broadens the available genetic base and illustrates the range of variation. The results of the present study showed that SCoT markers were efficient in assessing the genetic diversity among *E. sibiricus* accessions.

## 1. Introduction

*Elymus* L. is the largest genus of the tribe Triticeae and includes about 150 species worldwide [1]. *Elymus sibiricus* (Siberian wild rye) is one of the most important species of the genus. It is a perennial, self-pollinating and allotetraploid forage grass indigenous to northern Asia, but its geographic distribution extends from Sweden to Japan and even to parts of Alaska and Canada [2]. As a commercially useful species, as *E. sibiricus* is widely used in natural grassland and cultivated pastures due to excellent cold tolerance, good forage quality and adaptability, and it therefore plays an important role in Chinese animal husbandry and sustenance [3].

Genetic diversity is the foundation of species diversity and an important precursor in a study of any species, because its quantity and distribution has an effect on the evolutionary potential of species or populations [4]. Information on the genetic diversity and relationships among *E. sibiricus* accessions is limited but necessary for germplasm collection, conservation and breeding program. Several previous studies have examined *E. sibiricus* accessions and populations from Qinghai-Tibet Plateau [5,6,7] and worldwide [8] with the goal of improving the understanding of breeding materials. However, the genetic diversity and variation of most native *E. sibiricus* accessions from its primary distribution area including Qinghai-Tibet Plateau, Sichuan, Gansu, Inner Mongolia, and Xinjiang Provinces have not been well characterized. Thus, further analysis of the genetic diversity and variation of Chinese wild *E. sibiricus* accessions, cultivars and breeding lines from species range and their comparison may produce new insights and give a better understanding of the distribution of genetic diversity.

Traditional DNA markers have numerous applications in plant genetic diversity research. These markers include inter simple sequence repeat (ISSR) markers [5], sequence-related amplified polymorphism (SRAP) markers [6], and simple sequence repeat (SSR) markers [7] *etc.* In recent years, many new alternative and promising markers techniques have emerged. Start Codon Targeted (SCoT) polymorphisms are dominant and reproducible markers that are based on the short conserved region in plant genes surrounding the ATG translation start (or initiation) codon and use a single 18-mer primer in the polymerase chain reaction (PCR) assays and higher annealing temperature (50 °C) [9]. Markers are visualized by standard gel electrophoresis with agarose gels and staining making this technique suitable for the vast majority of plant research labs with standard equipment [9]. Lower recombination levels between SCoT markers and the gene/trait than random markers such as RAPDs, ISSRs or SSRs, making it possible to be used directly in marker-assisted breeding programs [10]. SCoT markers have been successfully used to assess genetic diversity and structure, identify cultivars, and for quantitative trait loci (QTL) mapping and DNA fingerprinting in different species, including rice, tritordeums, sugarcane, grape, potato, mango, myrica rubra, peanut, and garbanzo [9,11,12,13,14,15,16,17,18,19].

The present study examines the level of genetic diversity and its molecular variation of 53 *E. sibiricus* accessions from five geographic regions in China. The purposes of this study were: (a) to assess the genetic diversity and phylogenetic relationship among 53 *E*. s*ibiricus* accessions; and (b) to examine the effectiveness of the SCoT markers in *E. sibiricus* genetic diversity study. These results could facilitate *E. sibiricus* germplasm collection, conservation and future breeding.

## 2. Results and Discussion

### 2.1. Polymorphism of SCoT Markers

**Figure 1:**
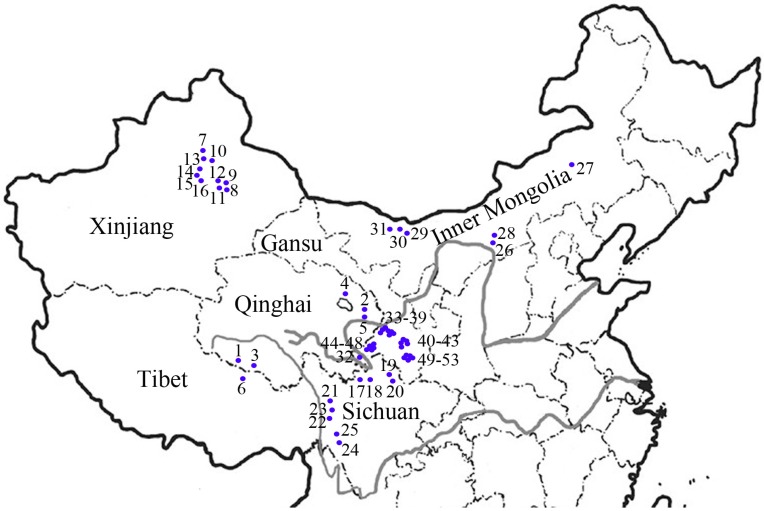
The geographic distribution of materials used in the study.

We analyzed the genetic diversity and variation of 53 *E. sibiricus* accessions from the species range in China (Table 1, Figure 1). A total of 173 bands were generated from 16 SCoT primers (Table 2). The total bands (TB) per primer ranged from six (SCoT30 and SCoT54) to 18 (SCoT23). The average band per primer was 10.8. The percentage of polymorphic bands (91.91%) was higher than the PPB values previous studies of SRAP variation (PPB = 86.48%) [6], SSR variation (PPB = 86.88%) [20], ISSR variation (PPB = 77.20%) [5], and RAPD variation (PPB = 78.65%) [21]. Previous studies showed that environment parameters are highly correlated with the magnitude and distribution of genetic diversity [5,8]. Compared with previous genetic diversity studies that mainly focused on populations or accessions from the Qinghai-Tibet Plateau, the present study had a wide geographical range. Thus, diverse geographical origin or ecological conditions may have contributed to the higher genetic diversity found among the *E. sibiricus* accessions tested. 

Polymorphic information content (PIC) varied from 0.20 (SCoT54) to 0.50 (SCoT41, SCoT42, SCoT62) with an average of 0.45 for this species. The resolving power (Rp) of the 16 SCoT primers ranged from 1.40 (SCoT54) to 6.49 (SCoT41). These primers (e.g. SCoT41, SCoT62) with higher PIC and Rp values have more potential for further study, allowing investigating more individuals or sampling sites with a reduced number of primers.

**Table 1:** *E. sibiricus* accessions used in the study.

POP	Code	Accession	Origin	Status
QX	1	Tongde	Qinghai, China	Cultivar
QX	2	PI504462	Qinghai, China	Wild
QX	3	Qingmu1	Qinghai, China	Cultivar
QX	4	PI504463	Qinghai, China	Wild
QX	5	PI531669	Qinghai, China	Wild
QX	6	PI639859	Tibet, China	Wild
XJ	7	PI499468	Xinjiang, China	Cultivated
XJ	8	PI499462	Xinjiang, China	Wild
XJ	9	PI619577	Xinjiang, China	Wild
XJ	10	PI595182	Xinjiang, China	Wild
XJ	11	PI499614	Xinjiang, China	Wild
XJ	12	PI499617	Xinjiang, China	Wild
XJ	13	PI499619	Xinjiang, China	Wild
XJ	14	PI595180	Xinjiang, China	Wild
XJ	15	PI655140	Xinjiang, China	Wild
XJ	16	Y2003	Xinjiang, China	Wild
SC	17	Y1005	Sichuan, China	Wild
SC	18	Chuancao2	Hongyuan, Sichuan, China	Cultivar
SC	19	Hongyuan	Hongyuan, Sichuan, China	Breeding line
SC	20	SAU133	Aba, Sichuan, China	wild
SC	21	SAU139	Kangding, Sichuan, China	Wild
SC	22	SAU003	Kangding, Sichuan, China	wild
SC	23	SAU137	Aba, Sichuan, China	Wild
SC	24	SC02	Ruoergai, Sichuan, China	Wild
SC	25	SC03	Ruoergai, Sichuan, China	Wild
NM	26	PI499457	Inner Mongolia, China	Cultivated
NM	27	PI499453	Inner Mongolia, China	Wild
NM	28	PI499456	Inner Mongolia, China	Cultivated
NM	29	PI499458	Inner Mongolia, China	Cultivated
NM	30	PI499459	Inner Mongolia, China	Cultivated
NM	31	W6 614214	Inner Mongolia, China	Cultivated
GS	32	MQ01	Maqu, Gansu, China	Wild
GS	33	HZ01	Hezuo, Gansu, China	Wild
GS	34	HZ02	Hezuo, Gansu, China	Wild
GS	35	HZ03	Hezuo, Gansu, China	Wild
GS	36	XH03	Xiahe, Gansu, China	Wild
GS	37	XH09	Xiahe, Gansu, China	Wild
GS	38	XH02	Xiahe, Gansu, China	Wild
GS	39	XH06	Xiahe, Gansu, China	Wild
GS	40	LT04	Lintan, Gansu, China	Wild
GS	41	LT02	Lintan, Gansu, China	Wild
GS	42	LT05	Lintan, Gansu, China	Wild
GS	43	LT01	Lintan, Gansu, China	Wild
GS	44	LQ01	Luqu, Gansu, China	Wild
GS	45	LQ03	Luqu, Gansu, China	Wild
GS	46	LQ04	Luqu, Gansu, China	Wild
GS	47	LQ09	Luqu, Gansu, China	Wild
GS	48	LQ10	Luqu, Gansu, China	Wild
GS	49	ZHN01	Zhuoni, Gansu, China	Wild
GS	50	ZHN05	Zhuoni, Gansu, China	Wild
GS	51	ZHN06	Zhuoni, Gansu, China	Wild
GS	52	ZHN03	Zhuoni, Gansu, China	Wild
GS	53	ZHN04	Zhuoni, Gansu, China	Wild

Note: QX = Qinghai-Tibet Plateau; XJ = Xinjiang, China; SC = Sichuan, China; NM = Inner Mongolia, China; GS = Gansu, China.

**Table 2:** 16 SCoT primers used to genotype *E. sibiricus* accessions, cultivars and breeding line, the total bands (TB), polymorphic bands (PB), percentage of polymorphic bands (PPB). Polymorphic information content (PIC), and resolving power of primer (Rp).

Primer ID	Primer Sequence (5'–3')	TB	PB	PPB(%)	PIC	Rp
SCoT 5	CAACAATGGCTACCACGA	10	9	90.00	0.45	2.65
SCoT 7	CAACAATGGCTACCACGG	7	6	85.7	0.41	2.53
SCoT 9	CAACAATGGCTACCAGCA	12	9	75.00	0.44	3.35
SCoT 10	CAACAATGGCTACCAGCC	13	11	84.62	0.45	2.40
SCoT 23	CACCATGGCTACCACCAG	18	18	100.00	0.47	5.20
SCoT 26	ACCATGGCTACCACCGTC	10	10	100.00	0.47	3.59
SCoT 30	CCATGGCTACCACCGGCG	6	5	83.33	0.46	2.71
SCoT 35	CATGGCTACCACCGGCCC	7	6	85.71	0.48	3.56
SCoT 41	CAATGGCTACCACTGACA	17	16	94.12	0.50	6.49
SCoT 42	CAATGGCTACCATTAGCG	11	10	90.91	0.50	2.56
SCoT 44	CAATGGCTACCATTAGCC	7	7	100.00	0.47	1.94
SCoT 45	ACAATGGCTACCACTGAC	11	7	63.64	0.45	2.14
SCoT 54	ACAATGGCTACCACCAGC	6	4	66.67	0.20	1.40
SCoT 60	ACAATGGCTACCACCACA	12	9	75.00	0.47	3.67
SCoT 61	CAACAATGGCTACCACCG	10	9	90.00	0.46	3.32
SCoT 62	ACCATGGCTACCACGGAG	16	14	87.50	0.50	3.86
Mean		10.8	9.4	89.60	0.45	---
Total		173	159	91.91	---	51.37

SCoT markers were used for the first time in *E. sibiricus* due to several advantages to other marker techniques: easier development of species-specific primers than SSR [22], lower cost than AFLP [22] and higher reproducibility than RAPD [18]. The results of the present study showed that SCoT markers were efficient in assessing the genetic diversity among *E. sibiricus* accessions.

### 2.2. Genetic Diversity Analysis

#### 2.2.1. Genetic Diversity among Regions

The NPB values ranged from 52 (NM) to 97 (QX), with an average of 65.8. The PPB values ranged from 30.06% (XJ) to 56.07% (QX), with an average of 37.63% (Table 3). The Shannon information index of diversity (I) values ranged from 0.1615 to 0.3002, with an average of 0.1983. The Nei’s genetic diversity (H) values ranged from 0.1068 to 0.2217, with an average of 0.1369. And the observed number of alleles values (Na) ranged from 1.3006 to 1.5797, with an average of 1.3841. This results was similar to previous reports of SRAP [6] (I = 0.2267, H = 0.1508) and SSR [20] (I = 0.1930, H = 0.1296), but lower than ISSR [5] (I = 0.269, H = 0.181) and RAPD [21] (I = 0.263, H = 0.176). Among the five geographical regions, the higher genetic diversity was observed in QX (NPB = 97; PPB = 56.07%; I = 0.3002; H = 0.2217; Na = 1.5797), followed by SC > GS > XJ > NM. Some factors could cause the differentiation of genetic variation of five geographical regions: climate conditions [23], topography [24], and sample size [25] *etc.* In many species, genetic variation is often positively associated with population size [25]. Different sample sizes of the five geographical regions may have an effect on measurement of genetic variation. However, eco-geographical factors may be more important than current sample size in determining patterns of diversity. Yan *et al.* [24] found that latitude, longitude and altitude are important factors that influence genetic difference of *Elymus* species. For example, accessions from the QX group originated from Qinghai and Tibet. The wider geographical range and diverse weather conditions may have contributed to higher estimate of genetic diversity among accessions in QX group. Therefore, in order to broaden the genetic base and sample the full extent of available variation, collecting and evaluating *E. sibiricus* germplasm from wide geographic regions and special eco-environment is important.

**Table 3:** Genetic variability within five geographic regions of *E. sibiricus* detected by SCoT markers.

POP	NPB	PPB (%)	I	H	Na
QX	97	56.07	0.3002	0.2217	1.5797
XJ	56	32.37	0.1662	0.1113	1.3237
SC	65	35.57	0.1978	0.1327	1.3757
NM	52	30.06	0.1657	0.1119	1.3006
GS	59	34.10	0.1615	0.1068	1.3410
Mean	65.8	37.63	0.1983	0.1369	1.3841

Note: NPB, number of polymorphic band; PPB, percentage of polymorphic bands; I, Shannon information index of diversity; H, Nei’s genetic diversity; Na, observed number of alleles.

#### 2.2.2. Genetic Diversity within Regions

AMOVA analysis showed that 50.99% of variation was apportioned within geographic regions and 49.01% was apportioned among geographic groups (Table 4). Similar patterns of genetic variation were found in wild *E. sibiricus* populations and other self-pollinating species such as *Elymus glaucus* and *Elymus trachycaulu* [26,27]. Previous researches using RAPD marker [21], ISSR marker [5], SRAP marker [6], and Gliadin [8] detected 59.95%, 57.52%, 65.29% and 63.4% genetic variation within *E*. *sibiricus* populations, respectively.

**Table 4:** Analysis of molecular variance (AMOVA) of five geographic regions.

Source of Variance	d.f.	Sum of Squares	Variance Component	Total Variation (%)
Among geographic regions	4	408.93	9.45	49.01
Within geographic regions	48	472.09	9.83	50.99

Previous reports revealed that self-pollinating species have relatively less within-population genetic variation than out-crossing species [28]. But other studies also demonstrated that gene mutation, gene flow, population size, sampling strategy can influence genetic variation [6,29,30,31]. In this study, accessions came from different geographic regions. Complex eco-geographical factors (e.g., intricate landforms and weather conditions) within the plant distribution may be related to genetic divergence within geographic regions, e.g., In Qinghai-Tibet Plateau, *E. sibiricus* germplasm are usually located in distant mountains, strongly isolated from each other by plateaus and valleys. These mountain and river valleys could serve as genetic barriers for pollinator movement and seed dispersal. The results of the present study suggested that more genetic variation of the species could be captured when sampling a larger number of plants from populations or geographic regions.

### 2.3. Population Structure and Cluster Analysis

**Figure 2:**
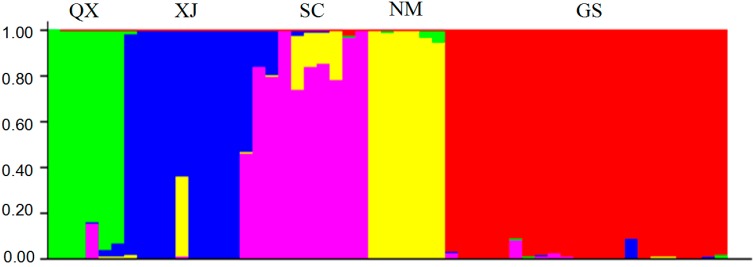
Five subgroups of 53 *E. sibiricus* accessions inferred from STRUCTURE analysis. The vertical coordinate of each subgroup indicates the membership coefficients for each accessions. Green zone: QX; Blue zone: XJ; Pink zone: SC; Yellow zone: NM; Red zone: GS.

The population structure of the 53 accessions was estimated using the Hardy-Weinberg Equilibrium by using STRUCTURE V2.3.4 software. Based on maximum likelihood and delta K (ΔK) values, the number of optimum groups was five (Figure 2). Among them, six accessions from Qinghai-Tibet Plateau were assigned to group 1 (QX); ten accessions from Xinjiang were assigned to group 2 (XJ); nine accessions from Sichuan were assigned to group 3 (SC); six accessions from Inner Mongolia were assigned to group 4 (NM); the rest twenty-two accessions from Gansu were assigned to group 5 (GS).

**Figure 3:**
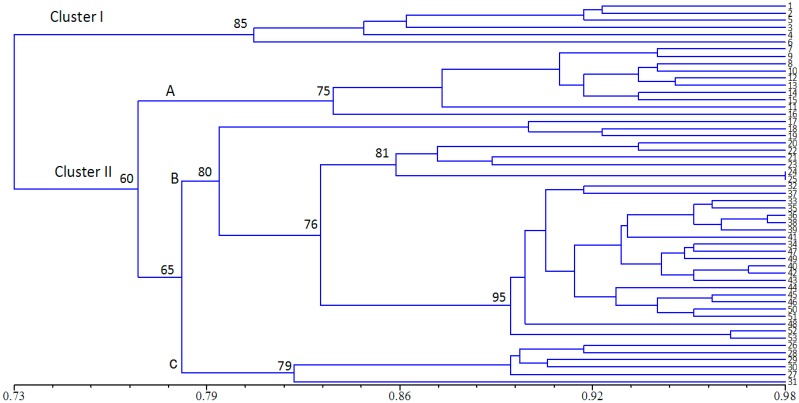
UPGMA-derived dendrogram of 53 *E. sibiricus* accessions based on Jaccard’s genetic similarity, only bootstrap values higher than 50% are presented. Accessions designations refer to Table 1.

**Figure 4:**
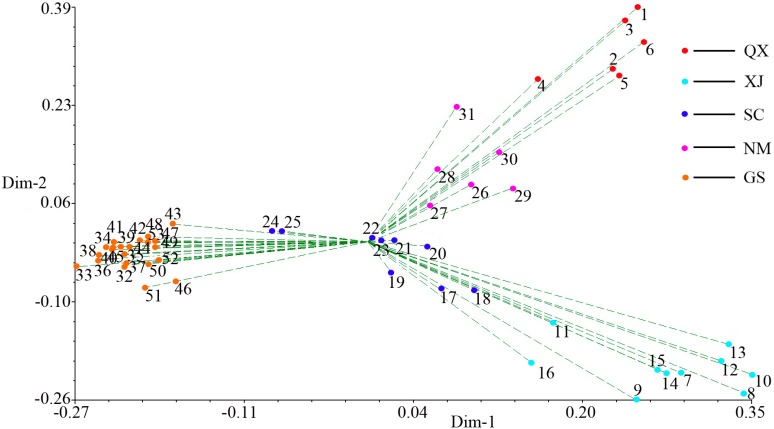
Principal coordinates analysis for the first and second coordinates estimated for SCoT markers using Jaccard’s genetic similarity matrix for 53 *E. sibiricus* accessions.

A dendrogram was constructed using data from UPGMA cluster analysis based on the genetic similarity matrix from all the accessions (Figure 3). The 53 accessions were clustered into two major clusters (with a similarity index of 0.73). Cluster I included 6 accessions collected from Qinghai and Tibet. Cluster II included 47 accessions: six from Inner Mongolia, nine from Sichuan, ten from Xinjiang, and twenty-two from Gansu. Furthermore, cluster II was classed into three sub-clusters. The first cluster (A) included all ten accessions from Xinjiang. The second cluster (B) included thirty one accessions collected from Sichuan and Gansu. The third cluster (C) included six accessions from Inner Mongolia. The dendrogram was almost in accordance with geographic origin of *E. sibiricus*, but this trend isn’t absolute. For example, nine Sichuan accessions were divided into two clusters. Six accessions were grouped with Gansu accessions with 76% bootstrap support. Ma *et al.* [5] evaluated eight *E. sibiricus* populations from the eastern Qinghai-Tibet Plateau regions using ISSR markers, which showed that there was no distinct geographical tendency in the distribution of the genetic diversity. Some geographically close accessions were clustered in different groups and geographically distant ones were clustered in the same groups. The various selection forces tend to produce genetic heterogeneity under the different small niches [32]. Moreover, the role of ecological factors in determining the extent and distribution of genetic diversity has been well documented [6,8,24]. Qinghai-Tibet Plateau is geographically isolated from other regions by towering mountains, which could result in the genetic difference between cluster I and cluster II. The relationship observed in the principal coordinate analysis (PCoA) was in agreement with the UPGMA analysis: about 42.43% of the total variation was described by the first three PCo, with PCo1 accounting for 21.83%, PCo2 for 12.00% and PCo3 for 8.60%, respectively (Figure 4). This multivariate approach was chosen to complement the cluster analysis information, because cluster analysis has a higher resolution for analysis of closely related populations, whereas the PCoA is more informative regarding distances among major groups. 

### 2.4. Conservation Implications

It is critical to understand the genetic diversity and variation among and within accessions to choose the effective strategy for conservation and sampling management. In this study, SCoT provided significant information on the genetic variation of *E. sibiricus* accessions and demonstrated an effective tool for the future tasks of genetic analysis, germplasm collection and conservation. The high degree of genetic variation and distinct geographical differentiation of *E. sibiricus* had been documented in this study. The ecological factors such as climatic types and eco-environment played a pivotal role in the divergence. Therefore, more attention should be paid to special eco-geographical groups (e.g., Qinghai-Tibet Plateau) with regard to the *E. sibiricus* germplasm collection and conservation.A major limitation of plant improvement programs is the lack of plant materials exhibiting rich genetic variation [33]. Past studies in wheatgrass [33] and orchardgrass [34] have demonstrated the importance of incorporation of useful genetic diversity into cultivars or cultivated materials. Based on Population structure and cluster analysis, most wild accessions from Xinjiang, Gansu and Inner Mongolia have been identified as genetically divergent to three *E. sibiricus* cultivars: “Tongde”, “Qingmu 1” and “Chuancao 2”. Thus, these accessions could be used as important genetic resources for genetic improvement of *E. sibiricus* in future breeding program.

## 3. Experimental Section

### 3.1. Plant Materials

A total of 53 *E. sibiricus* accessions were from the species range in China, comprising cultivars, breeding lines, cultivated and wild collections (Table 1). Materials were obtained from National Genetic Resources Program (USDA), the State Key Laboratory of Grassland Agro-ecosystems (Lanzhou University, China), Sichuan Agricultural University (Ya’an, China), and Sichuan Academy of Grassland Science (Sichuan, China). All accessions were grouped into five geographic regions: QX, XJ, SC, NM and GS based on their origin and physico-geographical regionalization. 

### 3.2. DNA Extraction

Twenty-five individuals of each accession were sampled for the extraction of bulk DNA. Plant tissues were collected from young seedlings, lyophilized, and used for DNA extraction using Tiangen plant genomic DNA kit, following the manufacturer’s protocol (Tiangen Biotech, Beijing, China). DNA quantity and quality were determined using a Nanodrop spectrophotometer (NanoDrop Products, Wilmington, DE, USA) and agarose gel electrophoresis.

### 3.3. SCoT-PCR Amplification

A total of 16 SCoT primers were randomly selected (Table 2). These primer sequences were published in previous studies [9,15]. Amplification was carried out in 15 μL samples containing 3 μL 25 ng/μL DNA, 7.5 μL 2× Reaction Mix (Tiangen), 0.15 μL 10 μM primer, 0.4 μL 2.5 U/μL Golden DNA Polymerase (Tiangen), and 3.95 μL of sterile ddH_2_O. PCR amplification was performed under the following conditions [22]: denaturation at 94 °C for 3 min, followed by 36 cycles of 94 °C for 1 min, 50 °C for 1 min, 72 °C for 2 min, and final extension at 72 °C for 5 min. The amplification products were separated in 1.6% agarose gels containing 0.12 μg/mL of Goldview through electrophoresis in 1X TBE buffer solution. The gel was photographed by a Gel Doc (TM) XR System (Bio-Rad, Hercules, CA, USA).

### 3.4. Data Analysis

The amplified bands were scored as presence (1) or absence (0), and only reproducible bands were considered. Polymorphic information content (PIC) values were calculated for each SCoT primers according to the formula: *PIC* = *1 − p2 − q2* [35]; where p is frequency of present band and q is frequency of absent band. The band informativeness (Ib) was calculated as *I_b_ = 1 − (2 × |0.5 − p|)* [36], where p is the proportion of the varieties or genotypes containing the band. The resolving power of the primer (Rp) was measured in accordance with Rp = ΣI_b_ [36]. The resulting present/absent data matrix was analyzed using POPGENE 32 Version 1.31 [37]. Number of polymorphic band (NPB), percentage polymorphic band (PPB), Shannon information index of diversity (I), Nei’s gene diversity (H), and observed number of alleles (Na) were calculated. The Analysis of Molecular Variance (AMOVA) was used to partition the total SCoT variation into within populations and among populations [38]. A dendrogram was constructed by Jaccard’s genetic similarity matrix to display accession relationships using the unweighted pair group method with arithmetic mean (UPGMA) of NTSYS (version 2.10) [39]. A principal coordinate analysis (PCoA) was constructed based on Jaccard’s genetic similarity matrix using DCENTER module in NTSYS. A bootstrap analysis with 1000 replicates was performed to obtain the confidence of branches of the cluster tree using the Winboot software [40]. Population structure of the 53 *E. sibiricus* accessions was analyzed using STRUCTRE v2.3.4 software [41] with the ‘admixture mode’, burn-in period of 10,000 iterations and a run of 100,000 replications of Markov Chain Monte Carlo (MCMC) after burn in. For each run, 10 independent runs of STRUCTURE were performed with the number of clusters (K) varying from 1 to 10. Maximum likelihood and delta K (△K) values were used to determine the optimum number of groups [41,42].The input files for POPGENE and AMOVA were prepared with the aid of DCFA1.1 program written by Zhang [43].

## 4. Conclusions

In the original research of Collard and Mackill [9] the use of SCoTs for genetic diversity assessment was suggested. However, SCoTs target potential coding genomic regions producing a dominant marker-system, but several co-dominant markers are also generated. Despite the high potential of SCoTs for targeted fingerprinting or QTL mapping purposes, those characteristics also consent their use for genetic diversity assessment [44]. The results of the present study showed that the highly reproducible SCoT markers were efficient in assessing the genetic diversity and relationships among *E. sibiricus* accessions.
